# Serum sodium variation is a major determinant of peridialytic blood pressure trends in haemodialysis outpatients

**DOI:** 10.1038/s41598-021-86960-2

**Published:** 2021-04-12

**Authors:** David A. Jaques, Andrew Davenport

**Affiliations:** 1 Division of Nephrology, Geneva University Hospitals, Rue Gabrielle-Perret-Gentil 4, 1205 Geneva, Switzerland; 2 UCL Department of Nephrology, Royal Free Hospital, University College London, London, UK

**Keywords:** Haemodialysis, End-stage renal disease

## Abstract

Intradialytic hypotension (IDH) and peridialytic blood pressure (BP) trends are associated with morbidity and mortality in haemodialysis (HD) patients. We aimed to characterise the respective influence of volume status and small solutes variation on peridialytic systolic BP (SBP) trends during HD. We retrospectively analysed the relative peridialytic SBP decrease in 647 prevalent outpatients attending for their mid-week session with corresponding pre- and post-HD bioelectrical impedance analysis. Mean SBP decreased by 10.5 ± 23.6 mmHg. Factors positively associated with the relative decrease in SBP were: serum sodium (Na) decrease, body mass index, serum albumin, dialysis vintage, ultrafiltration rate and urea Kt/V (*p* < 0.05 for all). Antihypertensive medications and higher dialysate calcium were negatively associated with the relative decrease in SBP (*p* < 0.05 for both). Age had a quadratic relationship with SBP trends (*p* < 0.05). Pre-HD volume status measured by extracellular to total body water ratio was not associated with SBP variation (*p* = 0.216). Peridialytic SBP trends represent a continuum with serum Na variation being a major determinant while volume status has negligible influence. Middle-aged and overweight patients are particularly prone to SBP decline. Tailoring Na and calcium dialysate concentrations could influence haemodynamic stability during HD and improve patient experience and outcomes.

## Introduction

More than 2 million patients with chronic kidney disease are treated with intermittent haemodialysis (HD) worldwide^1^. During a HD session, the systolic blood pressure (SBP) is expected to fall by 10 to 15 mmHg, mainly during the first hour of treatment^2^. While the definition of intradialytic hypotension (IDH) varies between studies and clinical guideline committees, it is the most commonly reported complication of outpatient HD treatments affecting up to 50% of ambulatory sessions and is associated with an increased risk of both patient morbidity and mortality^3,4^. Peridialytic blood pressure (BP) trends are also associated with cardiovascular and all-cause mortality^5^.

The underlying pathophysiology of BP regulation during HD is complex and multifactorial. During most HD sessions, fluid is removed from the vascular compartment by ultrafiltration (UF) to restore volume homeostasis. A decline in intravascular volume is expected to occur when the UF rate (UFR) exceeds the plasma refilling rate and UFR is thus regarded as an important determinant of IDH^6^. As such, patients with greater extracellular water (ECW) were reported to be at reduced risk of IDH^7^. On the other hand, intradialytic variation of small solutes could influence plasma refilling and patients starting HD with higher calculated osmolarity were reported to have greater falls in SBP^8^. Dialysate sodium (Na) manipulation and the fall of urea have also been associated with intradialytic hemodynamic stability^9,10^.

Although several individual factors have been reported to contribute to SBP changes during HD, their respective contributions and potential interplay in determining SBP trends have not been characterized. As clinical implications are potentially important, we aimed to describe the relationship between volume status, small solute variation and peridialytic SBP trends in a large HD outpatient cohort.

## Materials and methods

### Participants

We retrospectively reviewed the medical records of prevalent HD outpatients who attended for their mid-week HD session and had corresponding pre- and post-HD bioelectrical impedance analysis and laboratory evaluation as part of their routine follow-up. Patients were screened from two dialysis centres under the care of a single university hospital (Royal Free Hospital, University College London, London, UK) between January 2014 and December 2016. Patients were dialyzed with ultrapure quality water using Fresenius 5008 (Fresenius AG, Bad Homberg, Germany) or BBraun Dialogue + (BBraun, Melsungen, Germany) machines with high-flux polysulfone dialyzers (Fresenius AG, Bad Homberg, Germany) anticoagulated with low molecular weight heparin (Inhixa, Techdow Pharma Ltd, Guilford, UK). All dialysates contained magnesium 0.5 mEq/L, bicarbonate 28 mmol/L, acetate 3.0 mmol/L and glucose 5.5 mmol/L. Sodium dialysate concentration was at the discretion of the clinical care team, with a centre policy of using a dialysate sodium 136–138 mmol/L. Exclusion criteria were: (1) < 18 years-old, (2) limb amputation, or paralysis (3) presence of cardiac pacemaker or implantable defibrillator, and (4) recent urgent hospital admission. Patient co-morbidities and relevant medical history were obtained from computerized hospital medical records. Patients did not receive intravenous iron or erythropoietin-stimulating agents during the considered sessions.

### Variables

BP was measured in a standardized manner both immediately prior to and after the HD session in the non-fistula arm while in the sitting position with cuff size chosen according to patient’s arm size. BP was measured using the automated HD integrated monitors. Devices were regularly calibrated. Mean arterial pressure (MAP) was calculated as 1/3 * SBP + 2/3 * diastolic BP (DBP). SBP decrease was expressed in mmHg and defined as: pre-HD SBP–post-HD SBP. The decrease in SBP index was expressed as a percentage and defined as: (pre-HD SBP–post-HD SBP) / pre-HD SBP. DBP and MAP decrease as well as decrease in DBP and MAP index were defined similarly. Pre- and post-HD blood samples were measured using a standard multi-channel biochemical analyser (Roche Integra, Roche diagnostics, Lewes, UK) with an indirect ion-selective electrode technique for Na. Serum albumin was determined by the bromocresol green method. Total serum calcium was measured and ionized calcium calculated using the Mateu-de Antonio equation, derived from patients with kidney failure (0.815 × total calcium^0.5^)^11^. Bioelectrical impedance analysis was performed prior to and 20–30 min after the HD session using multifrequency segmental InBody 720 Body Composition Analysis (Biospace, Seoul, South Korea), following a standardized protocol^12^.

### Statistical analysis

Outliers were defined a priori when data collection error seemed more likely than true biological value with the following criteria: SBP < 40 or > 250 mmHg, DBP < 10 mmHg or > 200 mmHg, ECW / total body water ratio (ECW/TBW) < 35% or > 50%, serum Na < 100 mmol/L or > 160 mmol/L, potassium < 1 mmol/L or > 10 mmol/L, urea > 100 mmol/L and glucose > 60 mmol/L.

Continuous variables are expressed as mean ± standard deviation (SD) or median (interquartile range) according to distribution and categorical variables as number and relative frequencies. Normality of distribution was assessed graphically. In univariate analysis, patients were grouped into three categories based on the decrease in SBP: (1) hypotensive ≥ 20 mmHg (2) stable < 20 mmHg and > -− 10mmHg (3) hypertensive ≤ -− 10 mmHg. Patient’s characteristics were compared between groups using one-way ANOVA or Kruskal–Wallis and Chi2 for continuous and categorical variables respectively.

Multivariate linear regression with SBP decrease index as the dependent variable was used to build three distinct models adjusted for different covariates. In model 1, considered variables were: pre-HD ECW/TBW, Na decrease, potassium decrease, urea decrease, pre-HD glucose, age, gender, ethnicity (Caucasian vs non-Caucasian), body mass index (BMI), diabetes, antihypertensive medication, coronary artery disease and congestive heart failure. Model 2 was adjusted for the same variables as model 1 with the addition of: serum albumin, access type (arteriovenous fistula vs catheter) and dialysis vintage. Model 3 was adjusted for the same variables as model 2 with the addition of: dialysate calcium, dialysate temperature, UFR, session duration and sessional urea Kt/V. A final multivariate model was specified where variables that were not significant in model 3 were excluded using a stepwise backward procedure. Owing to a quadratic relationship with the decrease in SBP index, the square of age was considered in addition to age in every model.

As data could be collected on several occasions for every patient, multi-level mixed effect analysis was implemented for every regression model. For each model, linearity of relationship, normality of residuals and homoscedasticity of residuals were assessed graphically. Data were considered to be missing completely at random and therefore patients with any missing value were excluded from the multivariate analyses. Continuous variables were standardized to a mean of 0 and a SD of 1 in every model. A two-sided *p*-value < 0.05 was considered significant in every analysis. Statistical analyses were conducted using STATA version 15 (StataCorp, 4905 Lakeway Drive, College Station, Texas 77,845 USA).

### Ethical statement

Our retrospective audit was checked with, and complied with the United Kingdom (UK) National Health Service Health Research Authority guidelines for clinical audit and service development (https://www.hra.nhs.uk), and registered with the UCL Department of Nephrology Royal Free Hospital. All patient data was anonymized.

## Results

### Descriptive analysis

The entire cohort comprised 661 patients. As 14 patients were considered as outliers based on a priori specified criteria, 647 were included in the present analysis (Supplementary Figure S1).

Mean SBP decreased by 10.5 ± 23.6 mmHg, corresponding to a mean decrease in SBP index of 6.0 ± 16.1%. Mean DBP decreased by 3.2 ± 13.3 mmHg, corresponding to a mean decrease in DBP index of 2.3 ± 19.3%. Finally, mean MAP decreased by 5.6 ± 14.4 mmHg, corresponding to a mean decrease in MAP index of 4.7 ± 14.9%. Overall, 395 (61.0%) patients were men and median age was 66 (52–76). Patient demographic and clinical characteristics are described according to categories of SBP decrease in Table 1. Compared to stable and hypertensive patients, hypotensive patients were younger, had higher BMI, longer dialysis vintage, longer session duration, higher Kt/V, higher UFR, higher pre-HD serum ionized calcium, lower dialysate to serum calcium gradient, higher haemoglobin and higher albumin (*p* < 0.05 for all). Other considered characteristics were similar between groups.

**Table 1:** Patients characteristics according to peridialytic SBP trends.

Characteristics	Overall N = 647	Hypotensive N = 215	Stable N = 306	Hypertensive N = 126	*p* value
Pre-HD SBP (mmHg)	142.6 ± 27.2	156.9 ± 24.7	138.5 ± 25.7	128.3 ± 23.6	** < 0.001**
Post-HD SBP (mmHg)	132.1 ± 26.0	120.5 ± 21.2	132.8 ± 25.6	150.1 ± 23.9	** < 0.001**
SBP decrease (mmHg)	10.5 ± 23.6	36.3 ± 14.8	5.7 ± 8.0	− 21.8 ± 9.1	** < 0.001**
**Demographic characteristics**					
Age (years)	66 (52–76)	65 (53–76)	65 (51–74)	69.5 (56–79)	**0.046**
BMI (kg/m^2^)	26.2 ± 5.9	27.1 ± 6.2	26.0 ± 6.0	25.1 ± 5.2	**0.010**
Gender (men)	395 (61.0%)	125 (58.1%)	185 (60.4%)	85 (67.4%)	0.224
Race (Caucasian)	263 (41.1%)	82 (38.5%)	118 (39.3%)	63 (50.0%)	0.078
Antihypertensive medication	364 (56.7%)	110 (51.4%)	174 (57.4%)	80 (64.0%)	0.073
Diabetes	287 (44.6%)	102 (47.6%)	131 (43.2%)	54 (42.8%)	0.550
Congestive heart failure	78 (12.1%)	27 (12.6%)	34 (11.2%)	17 (13.4%)	0.774
Coronary artery disease	104 (16.1%)	39 (18.2%)	41 (13.5%)	24 (19.0%)	0.224
**Clinical characteristics**					
AVF	511 (79.4%)	175 (81.7%)	240 (78.9%)	96 (76.8%)	0.523
Vintage (months)	22.5 (9.2–60.2)	29.1 (13.3–71.6)	22.4 (8.7–55.2)	16.0 (5.4–44.1)	** < 0.001**
Session duration (hours)	3.74 ± 0.53	3.80 ± 0.52	3.76 ± 0.53	3.62 ± 0.54	**0.007**
Temperature (C°)	35.4 ± 0.4	35.4 ± 0.4	35.4 ± 0.4	35.4 ± 0.4	0.901
Qb (mL/min)	325 ± 35	328 ± 35	324 ± 35	322 ± 33	0.294
Kt/V	1.61 ± 0.38	1.67 ± 0.46	1.59 ± 0.35	1.54 ± 0.30	**0.021**
UFR (mL/kg/h)	6.75 ± 3.76	7.60 ± 4.12	6.30 ± 3.54	6.40 ± 3.38	**0.001**
Pre-HD dialysate sodium (mmol/L)	137.2 ± 1.4	137.1 ± 1.3	137.2 ± 1.3	137.3 ± 1.5	0.550
Post-HD dialysate sodium (mmol/L)	137.2 ± 1.3	137.1 ± 1.2	137.2 ± 1.3	137.2 ± 1.4	0.621
Dialysate calcium (mmol/L)	1.24 ± 0.16	1.24 ± 0.17	1.23 ± 0.17	1.27 ± 0.15	0.177
Serum ionized calcium^a^ (mmol/L)	1.23 ± 0.05	1.24 ± 0.05	1.23 ± 0.04	1.22 ± 0.05	**0.045**
Dialysate to serum calcium gradient (mmol/L)	0.009 ± 0.184	0.002 ± 0.188	0.001 ± 0.184	0.039 ± 0.175	**0.030**
Haemoglobin (g/L)	111.2 ± 13.3	113.3 ± 12.0	110.2 ± 13.9	109.9 ± 13.4	**0.016**
Albumin (g/L)	39.3 ± 4.2	39.7 ± 3.7	39.4 ± 4.1	38.2 ± 4.8	**0.032**

*SBP* systolic blood pressure, *BMI* body mass index, *AVF* arteriovenous fistula, *Qb* blood flow, *UFR* ultrafiltration rate, *HD* haemodialysis.

^a^Calculated as 0.815 * (total serum calcium)^0.5^.

Bold values correspond to *p* < 0.05.

Mean pre- and post-HD ECW/TBW were 40.0 ± 1.4% and 39.3 ± 1.6% respectively. Patient laboratory and bioelectrical impedance analysis characteristics are described according to categories of SBP decrease in Table 2. Compared to stable and hypertensive patients, hypotensive patients had higher pre-HD serum urea, greater urea decrease, lower pre- and post-HD ECW/TBW and higher body fat mass index (*p* < 0.05 for all). Other considered characteristics were similar between groups.

**Table 2:** Laboratory and bioelectrical impedance analysis characteristics.

Characteristics	Overall N = 647	Hypotensive N = 215	Stable N = 306	Hypertensive N = 126	*p* value
**Serum laboratory characteristics**					
Na pre-HD (mmol/L)	138.5 ± 3.4	138.5 ± 3.5	138.4 ± 3.4	138.5 ± 3.1	0.982
Na post-HD (mmol/L)	138.7 ± 2.3	138.6 ± 2.3	138.5 ± 2.4	139.1 ± 2.3	0.056
Na decrease (mmol/L)	− 0.2 ± 3.3	− 0.1 ± 3.3	− 0.1 ± 3.3	− 0.7 ± 3.3	0.241
K pre-HD (mmol/L)	5.0 ± 0.6	5.0 ± 0.7	5.0 ± 0.6	4.9 ± 0.7	0.123
K post-HD (mmol/L)	3.4 ± 0.4	3.4 ± 0.4	3.4 ± 0.4	3.4 ± 0.4	0.326
K decrease (mmol/L)	1.5 ± 0.6	1.6 ± 0.6	1.6 ± 0.6	1.5 ± 0.7	0.283
Urea pre-HD (mmol/L)	18.1 ± 5.6	18.9 ± 5.7	17.8 ± 5.5	17.4 ± 5.3	**0.024**
Urea post-HD (mmol/L)	4.4 (3.2–5.8)	4.4 (3.2–6.0)	4.4 (3.2–5.8)	4.4 (3.3–5.5)	0.888
Urea decrease (mmol/L)	13.3 ± 4.1	14.1 ± 4.2	13.0 ± 4.2	12.7 ± 3.9	**0.003**
Glucose pre-HD (mmol/L)	6.5 (5.4–7.8)	6.7 (5.5–8.3)	6.3 (5.4–7.5)	6.6 (5.5–7.8)	0.098
**Bioelectrical impedance analysis characteristics**					
ECW pre-HD (L)	14.9 ± 3.7	15.0 ± 3.6	14.9 ± 3.7	14.8 ± 3.5	0.929
ECW post-HD (L)	13.9 ± 3.5	13.8 ± 3.6	13.9 ± 3.6	13.9 ± 3.2	0.918
TBW pre-HD (L)	37.3 ± 9.3	37.4 ± 8.8	37.4 ± 9.5	37.0 ± 9.6	0.904
TBW post-HD (L)	35.3 ± 8.8	35.2 ± 8.6	35.5 ± 9.1	35.1 ± 8.2	0.896
ECW/TBW pre-HD (%)	40.0 ± 1.4	40.0 ± 1.6	40.0 ± 1.4	40.3 ± 1.3	**0.044**
ECW/TBW post-HD (%)	39.3 ± 1.6	39.3 ± 1.8	39.3 ± 1.6	39.6 ± 1.5	**0.034**
Smooth muscle mass index (kg/m^2^)	9.8 ± 1.7	9.9 ± 1.6	9.8 ± 1.8	9.6 ± 1.7	0.264
Body fat mass index (kg/m^2^)	8.4 ± 4.9	9.1 ± 5.3	8.3 ± 4.7	7.5 ± 4.5	**0.018**

Bold values correspond to *p* < 0.05.

*Na* sodium, *HD* haemodialysis, *K* potassium, *ECW* extracellular water, *TBW* total body water.

### Regression models

As 39 patients had missing values on considered covariates, 608 patients and 1′186 HD sessions were included in multivariate analysis. Median number of HD session per patient was 2 (1–2) with minimum and maximum values of 1 and 7 sessions respectively.

**Figure 1:**
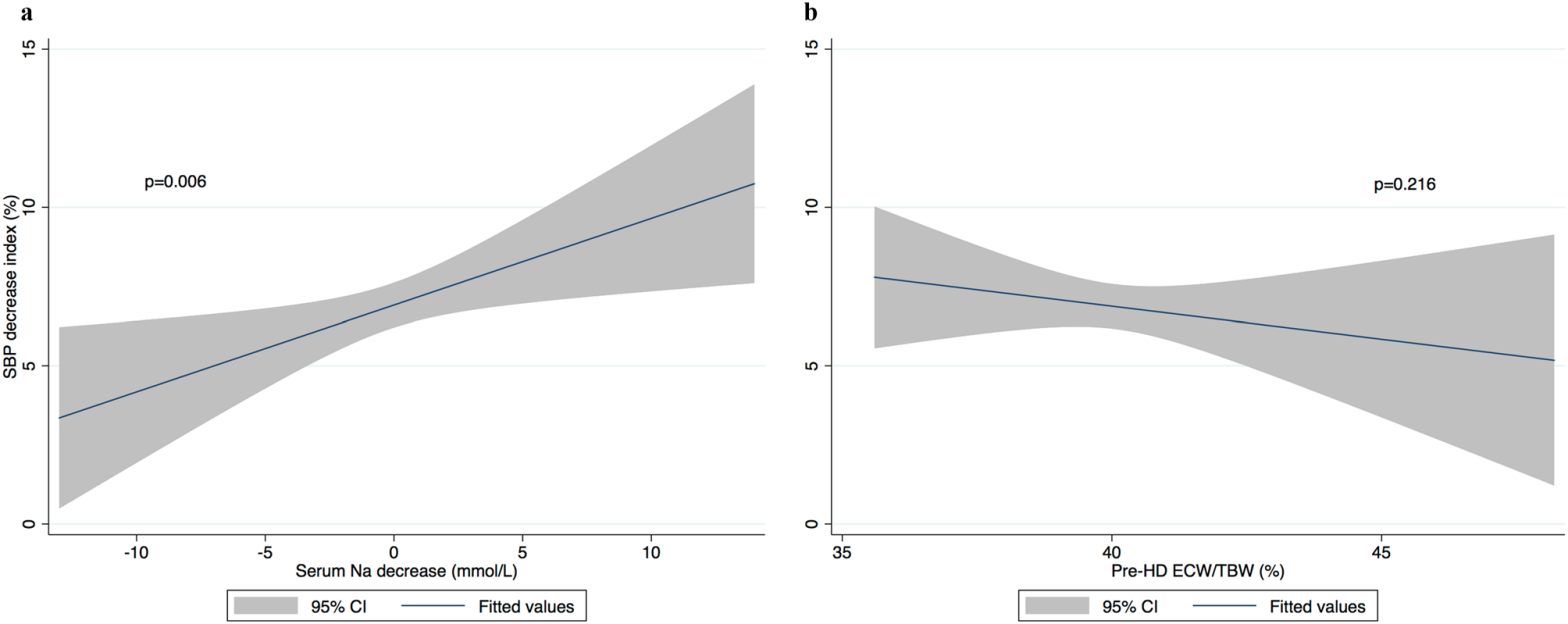
Multivariate relationship between SBP decrease index (%), serum Na decrease (mmol/L) and pre-HD ECW/TBW (%). (**a**) SBP decrease index (%) and serum Na decrease (mmol/L). (**b**) SBP decrease index (%) and pre-HD ECW/TBW (%). Relationship is based on the final multivariate model and is thus adjusted for: age, square of age, BMI, antihypertensive medication, serum albumin, dialysis vintage, dialysate calcium, UFR and Kt/V. Blue line represent fitted values and grey area represent 95% confidence interval. *SBP* systolic blood pressure, *Na* sodium, *HD* haemodialysis, *ECW* extracellular water, *TBW* total body water, *BMI* body mass index, *UFR* ultrafiltration rate.

**Figure 2:**
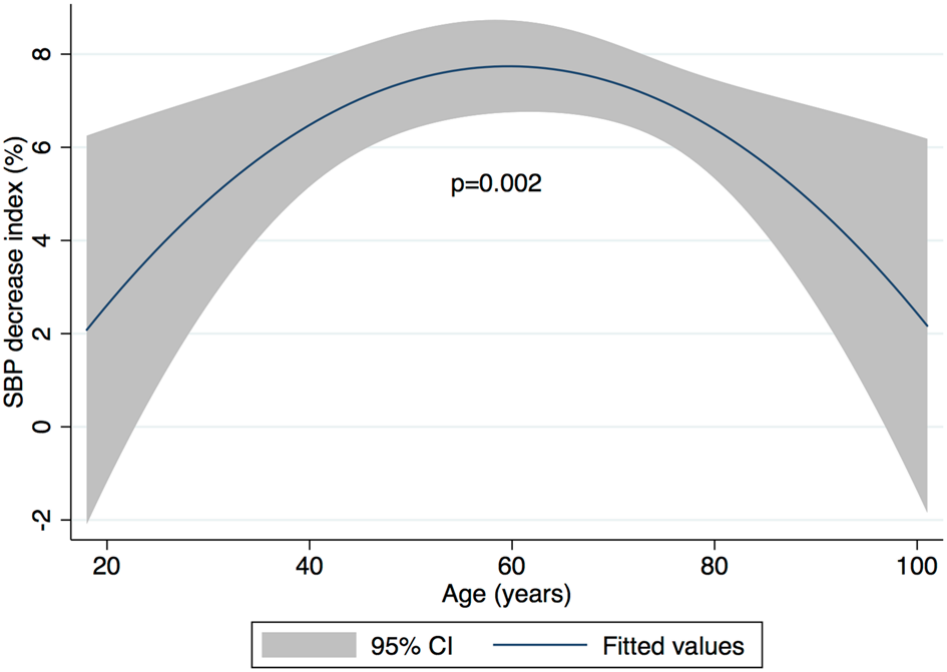
Multivariate relationship between SBP decrease index (%) and age (years). Relationship is based on the final multivariate model and is thus adjusted for: BMI, antihypertensive medication, serum albumin, dialysis vintage, dialysate calcium, UFR and Kt/V. Blue line represent fitted values and grey area represent 95% confidence interval. *SBP* systolic blood pressure, *BMI* body mass index, *UFR* ultrafiltration rate.

Serum Na decrease was positively associated with a decrease in SBP index in univariate analysis (*p* = 0.008) (Table 3). This association remained significant in all multivariate models (1, 2 and 3). Pre-HD ECW/TBW was negatively associated with a decrease in SBP index in univariate analysis (*p* = 0.005). This association however did not remain significant in multivariate models. In the final multivariate model, factors positively associated with a decrease in SBP index were: Serum Na decrease, BMI, serum albumin, dialysis vintage, UFR and Kt/V (*p* < 0.05 for all) (Table 4). Factors negatively associated with a decrease in SBP index were: antihypertensive medication and higher dialysate calcium (*p* < 0.05 for both). Based on standardized β coefficients, relative effect sizes of significant continuous variables in descending order were as follows: UFR, BMI, serum albumin, dialysis vintage, Kt/V, serum Na decrease and dialysate calcium. For every 1 SD (3.2 mmol/l) decrease in serum Na, there was a 1.19% decrease in SBP index (Fig. 1a). Pre-HD ECW/TBW was not associated with a decrease in SBP index (*p* = 0.216) (Fig. 1b). Age had a significant quadratic relationship with the decrease in SBP index (*p* = 0.002) (Fig. 2). During the stepwise backward procedure, non-significant variables were eliminated in the following order: access type, pre-HD glucose, congestive heart failure, dialysate temperature, decrease in serum potassium, ethnicity, decrease in serum urea, session duration, coronary artery disease, pre-HD ECW/TBW, gender and diabetes.

**Table 3:** Association of serum Na decrease (mmol/L) and pre-HD ECW/TBW (%) with SBP decrease index (%) in mixed linear regression.

	Serum Na decrease^a^ (mmol/L)		Pre-HD ECW/TBW^a^ (%)	
	β (95% CI)	*P* value	β (95% CI)	*P* value
Univariate	1.18 (0.30 to 2.05)	**0.008**	− 1.31 (− 2.23 to − 0.39)	**0.005**
Model 1	1.07 (0.18 to 1.95)	**0.017**	− 1.05 (− 2.12 to 0.01)	0.053
Model 2	0.98 (0.11 to 1.86)	**0.027**	− 0.62 (− 1.73 to 0.49)	0.276
Model 3	1.24 (0.37 to 2.11)	**0.005**	− 0.85 (− 1.96 to 0.25)	0.133

Model 1 is adjusted for: K decrease, urea decrease, pre-HD glucose, age, square of age, gender, ethnicity, BMI, diabetes, antihypertensive medication, coronary artery disease and congestive heart failure.

Model 2 is adjusted for same variables as model 1 as well as: serum albumin, presence of AVF and dialysis vintage.

Model 3 is adjusted for same variables as model 2 as well as: dialysate calcium, dialysate temperature, UFR, session time and Kt/V.

*Na* sodium, *HD* haemodialysis, *ECW* extracellular water, *TBW* total body water, *SBP* systolic blood pressure, *K* potassium, *BMI* body mass index, *AVF* arteriovenous fistula, *UFR* ultrafiltration rate.

^a^Standardized to a mean of 0 and a SD of 1.

Bold values correspond to *p* < 0.05.

**Table 4:** Factors significantly associated with SBP decrease index (%) in the final multivariate model.

Independent variables^a^	Final model	
	β (95% CI)	*P* value
Serum Na decrease	1.19 (0.34 to 2.05)	**0.006**
Age	− 1.33 (− 2.43 to − 0.22)	**0.018**
Age^2^	− 1.26 (− 2.05 to − 0.47)	**0.002**
BMI	1.99 (1.01 to 2.97)	** < 0.001**
Antihypertensive medication	− 1.89 (3.70 to − 0.09)	**0.039**
Serum albumin	1.66 (0.75 to 2.58)	** < 0.001**
Dialysis vintage^b^	1.24 (0.26 to 2.22)	**0.013**
Dialysate calcium	− 0.98 (− 1.86 to − 0.11)	**0.027**
UFR	2.13 (1.24 to 3.02)	** < 0.001**
Kt/V	1.21 (0.24 to 2.17)	**0.014**

*SBP* systolic blood pressure, *Na* sodium, *BMI* body mass index, *UFR* ultrafiltration rate.

^a^Standardized to a mean of 0 and a SD of 1.

^b^Log transformed.

Bold values correspond to *p* < 0.05.

### Sensitivity analyses

Absolute SBP decrease, instead of relative decrease in SBP index, was considered as the dependent variable in the final multivariate model. Results were similar and serum Na decrease remained positively associated with SBP decrease (*p* = 0.019) (Supplementary Table S1). Pre-HD ECW/TBW was not associated with a decrease in SBP (*p* = 0.414). Skeletal muscle mass index and body fat mass index, instead of BMI, were considered in the final multivariate model. Results were similar and the decrease in serum Na remained positively associated with the decrease in SBP index (β = 1.20, 95% CI 0.35 to 2.06, *p* = 0.006). Body fat mass index (β = 0.44, 95% CI 0.22 to 0.60, *p* < 0.001), but not skeletal muscle mass index (β = 0.41, 95% CI -0.14 to 1.03, *p* = 0.139), was positively associated with the decrease in SBP index. The final multivariate model was also adjusted for the administration of intradialytic IV fluid. Results were similar and the decrease in serum Na remained positively associated with the decrease in SBP index (*p* = 0.004) (Supplementary Table S2). Finally, DBP and MAP decrease index were considered as the dependent variables in final multivariate models instead of SBP decrease index. Serum Na decrease was not associated with the decrease in DBP index (eliminated during stepwise backward procedure with *p* = 0.237). Serum Na decrease was positively associated with the decrease in MAP index (β = 0.91, 95% CI 0.09 to 1.73, *p* = 0.029). Age had a significant quadratic relationship with the decrease in DBP and MAP index (*p* < 0.001 and *p* = 0.001 respectively).

## Discussion

Ultimately, BP regulation depends on cardiac output and systemic vascular resistance^13^. Fluid removal during HD is thus expected to influence BP with changes in preload and Nongnuch et al. observed that patients with greater extracellular volume, as measured by ECW/TBW, were less likely to experience a fall in SBP^7^. Likewise, the rapid decline in the concentration of small solutes in the intravascular compartment during HD would generate a temporary osmotic gradient potentially reducing plasma refilling and decreasing preload, and Mc Causland et al. recently reported that starting HD with higher calculated osmolarity, based on Na, urea and glucose, was associated with a greater decline in intradialytic SBP^8,14^. Rates of decline in urea during HD as well as the use of higher dialysate Na concentration or the administration of hyperosmolar substances may also promote intradialytic hemodynamic stability^9,10,15^. Compared to previous reports on changes in BP during HD, our study differs in two fundamental ways. First, in order to account for the intricate relationship between the variation in small solutes and volume status in determining hemodynamic stability, we measured both aspects concomitantly. Second, as the variation of such parameters during HD could be at least as important as their pre-HD state, we considered pre- and post-HD values. Using such a framework, we found that serum Na variation during HD was the major determinant of peridialytic SBP trends while volume status was not. Although this discordance with previous findings might theoretically be explained by a lack of statistical power to detect a small effect size of volume status on BP regulation, this hypothesis seems unlikely as previous studies reported significant results with smaller sample size^7^. Rather, our multivariate adjustment unveiled the importance of confounding factors, as pre-HD ECW/TBW was in fact associated with SBP trends prior to adjusting for relevant covariates. Analysing those parameters concomitantly is all the more important, in that variations in interdialytic weight gain and extracellular volume could be expected to influence measured concentrations of several small solutes such as Na and urea^16,17^. As such, Mc Causland et al. previously described a negative association between pre-HD Na and intradialytic SBP decline suggesting that lower serum Na would associate with greater interdialytic weight gain identifying patients who were more likely to have IDH as a result of higher UFR^8^. We demonstrate however, that serum Na variation is in fact an independent determinant of peridialytic SBP trends, even when accounting for volume status and prescribed UFR. The prime importance of serum Na variation rather than the pre-HD value is also highlighted by Mc Causland et al. as they noticed that pre-HD serum Na was no longer associated with intradialytic changes in SBP when patients with higher dialysate sodium were considered^8^. Although we found that serum Na variation had a major impact on SBP trends, urea did not. This once again illustrates the importance of multivariate adjustment, as we found that hypotensive patients had higher pre-HD urea and greater urea decrease compared to stable and hypertensive patients in univariate analysis. On a pathophysiological level, this implies that the contribution of individual small solutes should be considered rather that osmolarity calculated as a whole. Our findings are thus in agreement with urea being described as an ineffective osmole affecting osmolarity but not tonicity^18,19^.

Globally, our results thus indicate that intradialytic serum Na variation has important clinical implications for HD patients as it determines SBP trends independently of other important factors such as pre-HD volume status, session duration, UFR, Kt/V, dialysate temperature and administration of IV fluid during treatment. This supports the concept of improved hemodynamic stability associated with increased dialysate Na concentrations^20^. As our model allowed direct comparison of predictors’ relative effect sizes, it also becomes readily apparent that Na variation is one of the modifiable factors with the strongest impact on SBP trends beyond UFR. In our cohort, the observed range of Na variation could thus explain a difference of more than 7% in peridialytic SBP decrease.

While pivotal in SBP regulation, intradialytic serum Na variation did not influence peridialytic DBP trends in our study. Consequently, as MAP is entirely determined by SBP and DBP, Na variation had a significant although weaker impact on peridialytic MAP trends. Those findings are in agreement with a previous randomized trial on 10 HD patients reporting a significant influence of intradialytic serum Na variation on SBP but no such effect on DBP^21^. On a pathophysiological point of view, it is usually thought that SBP is mainly determined by stroke volume, while DBP is dependent on systemic vascular resistance^22,23^. As intradialytic variation of small solutes would be expected to influence plasma refilling, our results suggest that serum Na variation could impact preload, and thus stroke volume, leaving systemic vascular resistance unaffected.

Age was considered a risk factor of IDH in some studies^24,25^. Conversely, other reports found that older patients tended to increase their SBP during treatment^26^. Such a contradiction might arise when linearity assumptions are made on a non-linear association. It is thus striking that we found a clear quadratic relationship between age and SBP trends as it becomes apparent that each additional year has an opposite effect in younger compared to older patients, which would suggest that middle-aged patients could be more prone to IDH. An identical quadratic relationship was also found for DBP and MAP trends suggesting a global influence of age on BP regulation during dialysis. Lower body weight was reported to correlate with IDH in a previous study, but as absolute UF volumes were considered, then patients with lower body weight had relatively higher UFR^27^. In our study when accounting for relative UFR, we found the opposite, as patients with higher BMI were more likely to decrease their SBP during HD. This is however not unexpected as this additional weight is likely to reflect increased tissue mass rather than increased blood volume. Thus, a similar UFR would place a greater strain on the cardiovascular system of a heavier patients compared to a lighter one. In agreement with this hypothesis and with previous reports, we additionally showed that increased body fat mass favoured a decline in SBP during treatment^28^. Patients are frequently told to withhold their antihypertensive medication prior to dialysis to avoid the possibility of IDH^29^. Unfortunately, no study has clearly demonstrated that such a strategy is effective. Conversely, several interventional studies showed that prescription of antihypertensive agents could lower pre-HD BP without increasing the incidence of IDH^30–32^. Our results tend to support this hypothesis as the SBP decrease was less pronounced in patients prescribed antihypertensive drugs. Several studies reported interdialytic weight gain as a risk factor for IDH^6,31,33^. Although we did not formally measure interdialytic weight gain, ultrafiltration and session time are adequate proxies, both of which have been linked to increased mortality^34^. We found that UFR was the single most influential factor in determining SBP profile during HD. Our multivariate analysis also supports the important distinction that exists between interdialytic weight gain and absolute volume overload as parameters reflecting these two distinct concepts (UFR on one hand and ECW/TBW on the other) effectively showed differential effects on SBP trends^16^. Although not previously described, we report an association between elevated serum albumin and peridialytic decrease in SBP. Hypoalbuminemia in HD patients may be due to reduced synthesis and increased catabolism, both potentially driven by an inflammatory response^35,36^. However, it has also been suggested that lower albumin levels result from extracellular dilution in volume overloaded HD patients^37^. Thus, our results would support that higher albumin concentrations are associated with intravascular volume contraction, so increasing the risk of IDH. Finally, we observed that a lower dialysate calcium concentration was associated with peridialytic SBP decline. This result supports several earlier smaller studies which have reported greater falls in SBP with lower calcium dialysates, in particular in combination with low magnesium dialysates^38–40^. Concerns that using higher dialysate calcium concentrations may promote a positive calcium balance have led to an increase use of lower calcium containing dialysates^41^. As such, we noted that patients with higher pre-HD serum calcium were prescribed lower calcium dialysate concentration resulting in a lower dialysate to serum calcium gradient ultimately favouring peridialytic BP decline.

As with any observational study, causal inference is inherently limited and confounding by indication has to be considered. However, as most included variables were not readily modifiable and several potential confounders were included, our conclusions should not be significantly altered. Intradialytic BP measurements were not available in our study. Our goal was however slightly different as we considered dialytic SBP regulation as a continuous phenomenon rather than focusing on an arbitrary definition of IDH. As such, the observed linearity of the relationship between SBP variation and its clinical determinants highlighted the fact that quantitative, and not qualitative, differences physiologically determined hemodynamic stability during HD. Likewise, ambulatory interdialytic BP measurements were not considered in our study. While interdialytic BP values are of greater prognostic significance regarding end-organ damage and cardiovascular outcomes, they do not reflect the impact of dialysis itself on hemodynamic regulation^42^. Post-HD glucose was not analysed as it is not routinely measured at our institution. However, pre-HD glucose showed no association with our main outcome and was among the first discarded variables during the backward stepwise procedure. Thus, as all patients were dialyzed against a standard 5.5 mmol/L glucose dialysate, we have no reason to believe that post-HD glucose would have been associated with our outcome. Even so, our main conclusions would likely not have been affected by such an adjustment.

## Conclusion

In this observational study, we present novel insights into the hemodynamic regulation during HD by describing clinical determinants of peridialytic BP patterns in a large HD outpatient population. We found that serum Na variation is a major determinant of SBP regulation during HD while volume status at the start of the treatment only plays a negligible role when accounting for relevant clinical variables. While younger as well as older patients tended to maintain their BP throughout treatment, middle-aged and overweight individuals were prone to peridialytic BP decline. Elevated serum calcium also favoured hypotension by clinicians choosing lower dialysate calcium, so decreasing dialysate to serum calcium gradient. In light of these findings, peridialytic BP trends should be perceived as part of a quantitative spectrum determined by certain modifiable physiological variables. Tailoring Na and calcium dialysate prescription could potentially influence hemodynamic stability during HD regardless of volume status and improve patient experience and outcomes.

## Supplementary Information

Supplementary Information

## Data Availability

Data supporting the findings of this study are available from the corresponding author upon reasonable request.
