# Supplementary material for: Serum sodium variation is a major determinant of peridialytic blood pressure trends in haemodialysis outpatients
Source: Sci Rep. 2021 Apr 12;11:7882. doi: 10.1038/s41598-021-86960-2 (PMC8042038; doi:10.1038/s41598-021-86960-2)
Supplement: Supplementary file 1 — Supplementary Information [file 41598_2021_86960_MOESM1_ESM.pdf]

SERUM SODIUM VARIATION IS A MAJOR DETERMINANT OF PERIDIALYTIC BLOOD PRESSURE TRENDS IN HAEMODIALYSIS OUTPATIENTS.

David A. JAQUES; Andrew DAVENPORT.

SUPPLEMENTARY MATERIAL

Supplementary figure S1: Study flowchart

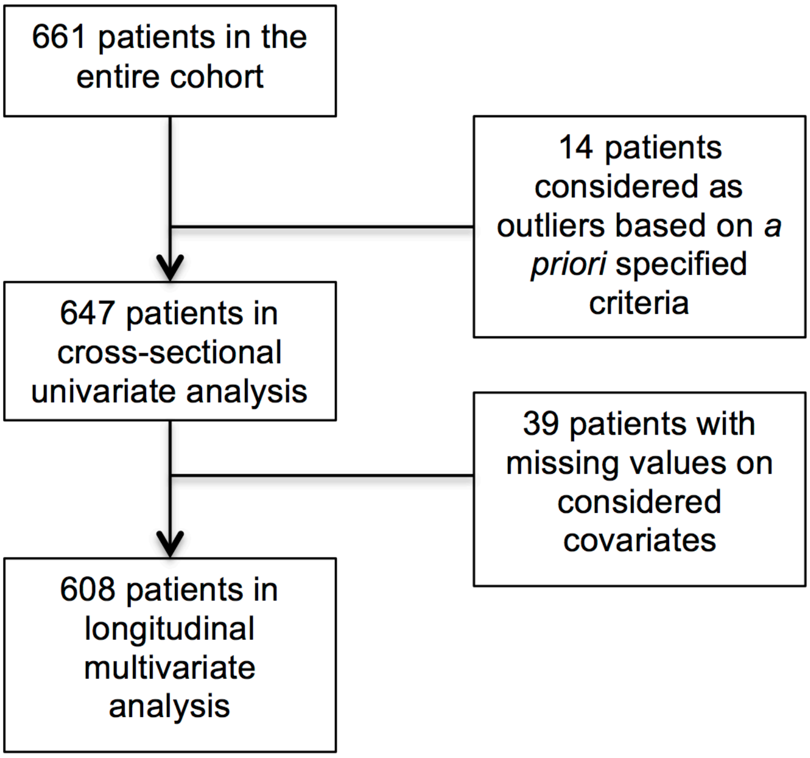

Supplementary table S1: Factors associated with SBP decrease (mmHg) in the final multivariate model.

**Independent variables**[a]	**Final model**	
	**β (95% CI)**	**P value**
**Serum Na decrease**	1.51 (0.24 to 2.77)	**0.019**
**Age**	-1.42 (-3.06 to 0.21)	0.089
**Age^2**	-1.71 (-2.87 to -0.54)	**0.004**
**BMI**	3.04 (1.60 to 4.49)	**<0.001**
**Antihypertensive medication**	-2.47 (-5.15 to 0.19)	0.069
**Serum albumin**	2.16 (0.80 to 3.52)	**0.002**
**Dialysis vintage**[b]	1.58 (0.13 to 3.03)	**0.032**
**Dialysate calcium**	-1.66 (-2.96 to -0.37)	**0.012**
**UFR**	3.26 (1.94 to 4.57)	**<0.001**
**Kt/V**	1.61 (0.18 to 3.03)	**0.026**

a: Standardized to a mean of 0 and a SD of 1.

b: Log transformed.

Bold values correspond to p<0.05.

Abbreviations: SBP, systolic blood pressure; Na, sodium; BMI, body mass index; UFR, ultrafiltration rate.

Supplementary table S2: Factors associated with SBP decrease index (%) in the final multivariate model adjusted for the administration of intradialytic IV fluid.

**Independent variables**[a]	**Final model**	
	**β (95% CI)**	**P value**
**Serum Na decrease**	1.26 (0.40 to 2.12)	**0.004**
**Age**	-1.35 (-2.47 to -0.22)	**0.018**
**Age^2**	-1.29 (-2.09 to -0.50)	**0.001**
**BMI**	1.93 (0.95 to 2.92)	**<0.001**
**Antihypertensive medication**	-2.11 (-3.94 to -0.28)	**0.024**
**Serum albumin**	1.60 (0.67 to 2.53)	**0.001**
**Dialysis vintage**[b]	1.35 (0.36 to 2.35)	**0.007**
**Dialysate calcium**	-1.01 (-1.89 to -0.14)	**0.023**
**UFR**	2.31 (1.40 to 3.23)	**<0.001**
**Kt/V**	1.19 (0.21 to 2.17)	**0.017**
**Intradialytic IV fluid**	5.86 (0.85 to 10.86)	**0.022**

a: Standardized to a mean of 0 and a SD of 1.

b: Log transformed.

Bold values correspond to p<0.05.

Abbreviations: SBP, systolic blood pressure; Na, sodium; BMI, body mass index; UFR, ultrafiltration rate.
